# Comparative Genomics Study of Multi-Drug-Resistance Mechanisms in the Antibiotic-Resistant *Streptococcus suis* R61 Strain

**DOI:** 10.1371/journal.pone.0024988

**Published:** 2011-09-26

**Authors:** Pan Hu, Ming Yang, Anding Zhang, Jiayan Wu, Bo Chen, Yafeng Hua, Jun Yu, Huanchun Chen, Jingfa Xiao, Meilin Jin

**Affiliations:** 1 Division of Animal Infectious Disease in the State Key Laboratory of Agricultural Microbiology, College of Veterinary Medicine, Huazhong Agricultural University, Wuhan, People's Republic of China; 2 CAS Key Laboratory of Genome Sciences and Information, Beijing Institute of Genomics, Chinese Academy of Sciences, Beijing, People's Republic of China; 3 Graduate University of Chinese Academy of Sciences, Beijing, People's Republic of China; Emory University, United States of America

## Abstract

**Background:**

*Streptococcus suis* infections are a serious problem for both humans and pigs worldwide. The emergence and increasing prevalence of antibiotic-resistant *S. suis* strains pose significant clinical and societal challenges.

**Results:**

In our study, we sequenced one multi-drug-resistant *S. suis* strain, R61, and one *S. suis* strain, A7, which is fully sensitive to all tested antibiotics. Comparative genomic analysis revealed that the R61 strain is phylogenetically distinct from other *S. suis* strains, and the genome of R61 exhibits extreme levels of evolutionary plasticity with high levels of gene gain and loss. Our results indicate that the multi-drug-resistant strain R61 has evolved three main categories of resistance.

**Conclusions:**

Comparative genomic analysis of *S. suis* strains with diverse drug-resistant phenotypes provided evidence that horizontal gene transfer is an important evolutionary force in shaping the genome of multi-drug-resistant strain R61. In this study, we discovered novel and previously unexamined mutations that are strong candidates for conferring drug resistance. We believe that these mutations will provide crucial clues for designing new drugs against this pathogen. In addition, our work provides a clear demonstration that the use of drugs has driven the emergence of the multi-drug-resistant strain R61.

## Introduction

*Streptococcus suis* is an emerging zoonotic pathogen with a worldwide distribution. This pathogen causes meningitis, endocarditis, septicemia, septic arthritis, pneumonia, and abortion in pigs [1]. In the swine industry, *S. suis* is one of the major causes of bacterial infections, and these infections can cause considerable economic loss. Human cases are usually described as occupational infections that result from direct contact with infected pigs or pig products, but sudden outbreaks have recently been reported [2]. Human deaths have often been caused by meningitis, but many could be attributed to a novel form of invasive toxic shock syndrome [3]. Concomitant with the increasing prevalence of *S. suis* infections, multi-antimicrobials have been copiously used against bacterial pathogens. However, resistance to antimicrobials, including tetracycline, macrolide-lincosamide-streptogramin B (MLSB) and fluoroquinolones, has been widely reported in *S. suis* strains isolated from pigs [4]–[10]. As resistance becomes more common, there is a greater need for alternative treatments. Despite a push for new antibiotic therapies, there has been a continued decline in the number of newly approved drugs [11]. Therefore, the emergence of antibiotic-resistant strains poses an intricate problem for both pig production and public health [12]–[14].

Antimicrobial susceptibility profiles and the corresponding resistance determinants of *S. suis* have been extensively reported. However, the genome dynamics and molecular basis for the mechanism of drug resistance of *S. Suis* are still not well explained because of a lack of pertinent sequence information. In the past few years, the development of next-generation massively parallel sequencing technologies has significantly improved sequencing throughput while simultaneously reducing costs [15]. Numerous bacteria have complete genome sequence data available on public databases. Multiple genome sequences from different strains of a single species offer comprehensive information for exploring the relationship between genotypes and phenotypes, further determining the genetic basis of biological functions and illustrating the mechanisms of evolution. To investigate the associations between drug-resistance mechanism and genetic components diversity of *S. suis*, we sequenced two *S. suis* strains R61 (from the lung of a diseased pig) and A7 (from the brain of a diseased pig). Of the currently sequenced *S. suis* isolates, strain R61 is resistant to most antibiotics, whereas strain A7 is fully sensitive to all tested antibiotics. Determining differences in genes' components between the high and low drug-resistant strains would enable us to understand the pattern of drug-resistant gene movement between organisms and the mechanisms of drug-resistance. R61 and A7 genomes were analyzed in comparison with the complete genome sequences of *S. suis* strains P1/7, SC84, and BM407. This comparative genomic analysis allowed us to characterize this pathogenic *S. suis* strain on a global genomic scale.

## Results and Discussion

### Antimicrobial susceptibility profiles

The results of minimum inhibitory concentration (MIC) testing indicated that R61 and A7 isolates have almost completely different antimicrobial susceptibility profiles. *S. suis* strain A7 was fully susceptible to all 18 antibiotics tested (Table 1). However, *S. suis* strain R61 was resistant to 15 antibiotic agents, including ampicillin, penicillin, cefotaxime, ceftriaxone, cefuroxime, cefaclor, azithromycin, erythromycin, tetracycline, chloramphenicol, clindamycin, levofloxacin, gatifloxacin, amoxicillin with clavulanate potassium and trimethoprim. Strain R61 was susceptible to cefepime, meropenem and vancomycin. Two previous studies have examined the antimicrobial susceptibility phenotypes of the *S. suis* P1/7, SC84 and BM407 [16], [17] strains. Antimicrobial susceptibility profiles of these five strains are listed in Table 2. Of the 18 antibiotics used in our MIC test, strain SC84 is resistant to tetracycline, and strain P1/7 is resistant to erythromycin, azithromycin, tetracycline and chloramphenicol. Strain BM407 is resistant to erythromycin, azithromycin, tetracycline and chloramphenicol, but it is susceptible to penicillin and cefuroxime (Table 2).

**Table 1:** MICs of 18 antimicrobial agents for *S. suis* A7 and R61 isolated from pigs in China (µg/ml).

Strains	Antibiotics MIC (mg/ml)																	
	AMP	PEN	CPE	CFT	CAX	CRM	CFR	AZI	ERY	TET	CHR	CLI	LVX	GAT	MER	VAN	AUG	TSZ
ATCC49619	≤0.06	0.25	≤0.25	≤0.25	≤0.25	0.5	1	≤0.25	≤0.06	≤0.5	2	≤0.06	0.5	0.12	0.12	≤0.12	≤0.5/0.25	≤0.25/4.75
A7	≤0.06	≤0.03	≤0.25	≤0.25	≤0.25	≤0.25	≤0.5	≤0.25	≤0.06	1	4	≤0.06	0.75	0.25	≤0.06	0.25	≤0.5/0.25	≤0.25/4.75
R61	>4	>4	2	>2	>2	>2	>4	>2	>0.5	>4	>16	>0.5	>32	32	0.12	0.25	>4/2	>2/38

Abbreviations: AMP, ampicillin; PEN, penicillin; CPE, cefepime; CFT, cefotaxime; CAX, ceftriaxone; CRM, cefuroxime; CFR, cefaclor; AZI, azithromycin; ERY, erythromycin; TET, tetracycline; CHR, chloramphenicol; CLI, clindamycin; LVX, levofloxacin; GAT, gatifloxacin; MER, meropenem; VAN, vancomycin; AUG, Amoxycillin with clavulanate potassium; TSZ, trimethoprim/sulfamethoxazole;

**Table 2:** Summary of antimicrobial resistance of *S. suis* isolates.

Resistance phenotype	R61	A7	P1/7	SC84	BM407
PEN	+	−	−	−	−
AMP	+	−	−	−	ND
AMC	+	−	ND	ND	ND
CXM	+	−	ND	ND	ND
CEC	+	−	ND	ND	ND
CTX	+	−	ND	−	ND
CRM	+	−	ND	−	−
LVX	+	−	ND	−	ND
GAT	+	−	ND	ND	ND
ERY	+	−	−	−	+
AZI	+	−	ND	−	+
TET	+	−	−	+	+
CLI	+	−	ND	ND	ND
CHR	+	−	−	−	+

ND, not determined. The plus sign means resistance. Conversely, the minus sign stands for sensitivity. The antibiotic abbreviations are shown in Table 1. The information of resistance phenotype of P1/7, SC84 and BM407 comes from published papers [16], [17].

### General features of sequenced genomes

We generated 2.9 billion high-quality base pairs for a 1,247-fold genome coverage of R61 and 1.4 billion high-quality base pairs for a 687-fold genome coverage of A7. The total length of the assembled R61 genome is 2,390,900 bp, made up of 53 contigs. The N50 contig size is 24,004 bp. The genome of A7 consists of a single circular chromosome of 2,038,409 bp (Figure S1; accession number CP002570). No plasmids were detected in the assembly or by gel electrophoresis experiments using a 1 kb DNA marker, which were performed to estimate sample purity prior to genome sequencing. In total, 2,346 coding sequences (CDSs) were identified in the R61 genome. This is obviously more sequences than in any other previously sequenced *S. suis* strain. However, the rRNA and tRNA cluster copy numbers were both fewer than in other *S. suis* strains, which may help explain why the repeats were masked during scaffold construction. Large-scale repeats are always difficult in genome assembly, and it is hard to determine the real copy number of repeat regions before completely sequencing the genome. Therefore, the rRNA copy number presented in Table 3 is only the outcome of the scaffold sequences, not a real reflection of the final complete genome. The copy number of rRNA clusters in R61 currently remains uncertain. The number of insert sequence (IS) elements regions identified in R61 is slightly greater than in other *S. suis* strains. IS elements encode only the functions involved in their translocation and transposition, both within and between genomes. IS elements are thought to be one of the major players in prokaryote genome [18] plasticity. A greater number of IS elements indicates that the genome has undergone more structural variations during strain evolution. To investigate conservation of sequence blocks between strains R61 and A7, we used the Mummer 3 software package [19] to map R61 contig sequences to the A7 finished genome (Figure S2). We determined that there are multi-locus genome variations, including duplications, inversions and deletions, between the two genomes.

**Table 3:** General features of *S. suis* genome.

Characteristic	Description or value for strain				
	R61	A7	P1/7	SC84	BM407
Source(Location)	Pig(China)	Pig(China)	Pig(European)	Human(China)	Human(Vietnam)
Culture data	2010	2010	2009	2005	2004
Serotype	ND	2	1	7	1
Size(bp)	∼2,390,900	2,038,409	2,007,491	2,095,898	2,146,229
G+C content (%)	41.2	41.2	41.3	41.1	41.1
No. of CDSs	2346	1974	1908	1985	2040
Coding density (%)	86.2	88.7	85.1	84.8	83.9
Avg. length of CDSs (bp)	850	887	931	933	932
rRNA (16S-23S-5S)	3	4	4	4	4
tRNA	46	56	56	56	56
IS elements	34	25	27	28	32
Reference(s)	This study	This study	[16]	[16]	[16]

Predicted amino acid sequences of each strain were compared with the Antibiotic Resistance Genes Database (ARDB) [20] to preliminarily scan for drug-resistant genes in these strains. The results are shown in Table S1. Each strain seems to possess one bacitracin-resistant gene. The table demonstrates that strains A7 and P1/7 have the weakest drug-resistant phenotype, and R61 shows the strongest drug-resistant profile among the studied *S. suis* strains. There were three *tet* genes and one *erm* gene detected in BM407 and only one *tet* gene in SC84, which is consistent with previously reported results [16], [17].

### Antimicrobial resistance patterns

The 18 antibiotics used in our test are conventionally categorized into several types based on their mechanism of action, chemical structure, or spectrum of activity. For example, ampicillin, amoxicillin-clavulanic acid, cefuroxime and cefotaxime are grouped as β-lactam antibiotics. Levofloxacin and gatifloxacin are known as quinolones. Erythromycin and azithromycin are macrolides. Our analysis reveals that R61 resistance to the 15 tested antibiotics tested is mainly derived from 3 classes of action: alteration in target site, target protection and reduced drug accumulation.

### Pattern 1: Alteration of the target site to give resistance against β-lactam and quinolone antibiotics

Altering target sites is the main strategy of bacteria for developing resistance against antibacterial drugs. Currently, β-lactam antibiotics are the most widely utilized antibiotics owing to their comparatively high effectiveness, low cost, ease of delivery and minimal side effects [21]. Resistance to β-lactam antibiotics has been widely reported in *Streptococcus pneumoniae*. In contrast, knowledge regarding resistance to β-lactam antibiotics in *S. suis* is very limited. β-lactam resistance in *S. pneumoniae* is a consequence of generating mosaic *pbp* genes. Such genes encode penicillin-binding proteins (PBPs) harboring tens of substitutions throughout the entire protein [22], which causes these genes to lose their affinity for the antibiotics.

**Figure 1:**
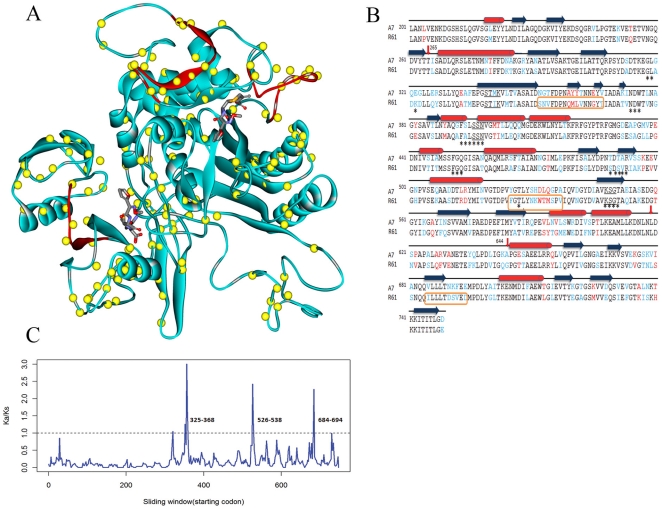
Comparison of PBP2x-A7 and PBP2x-R61. (A). Structure of PBP2x from drug-resistant strain R61. Yellow spheres represent locations of mutations (as compared with the PBP2x sequence from the penicillin-sensitive strain A7). Two molecules of cefuroxime are placed in an equivalent position to that observed in the complex from PDB 1QMF. Three detected positive selection regions are colored in red. The result demonstrates that the three regions are all located in loops. Two of the three regions are adjacent to the upper cefuroxime molecule and surround the cleft of catalytic active site. The third positive selection region is far away from the active site and only located in the C-terminal domain. However, this region is very close to the lower cefuroxime molecule. (B). Sequence alignment of PBP2x-A7 and PBP2x-R61 and secondary structure assignment. Conserved mutations are represented in blue, and non-conserved changes are shown in red. Secondary structural elements in the PBP2x-R61 structure are shown as red cylinders (α-helices) and blue rectangles (β-sheets). Sequence motifs relevant for catalysis (SXXK, SXN and KSG) are underlined. The transpeptidase domain consists of residues 256–619. Residues that are less than 5 Å from the active site are marked with asterisks. Residues in regions that are under positive selection are shown within orange rectangular boxes. (C). Selection pressure on the PBP2x-R61 sequence. We used a sliding window of 10 codons (step size 2 codons) along the PBP2x-R61 DNA sequence to expose selective pressures on different regions. In the plot, although the general trend is below the threshold of 1 (indicated by a dotted line), there are 3 peaks over the threshold, indicating that these regions (residues 325–368,526–538 and 684–694) are under positive selection. Mutations in these regions appear to provide some fitness advantage to the bacterial strain.

Four PBPs were identified and classified on the basis of their sequence similarities in R61 and A7. These are *pbp2x*, *pbp2b*, *pbp1a* and *pbp2a*. However, the major contributors to *S. suis* β-lactam resistance were still unclear. To determine whether the resistance mechanism in *S. suis* was the same as in *S. pneumoniae*, we aligned the four kinds of PBP sequences from strains R61, A7, P1/7, BM407 and SC84. The latter four strains are all β-lactam sensitive. Comparing the sequences demonstrated that the PBP sequences are highly homologous in the four β-lactam sensitive strains. However, each of the four PBP genes in R61 genome harbors multiple substitutions throughout entire sequence. PBP2x, which is described as the primary PBP target in β-lactam-resistant *S. pneumoniae* strains, appears the most percentage of substitutions among the four PBPs in R61 genome as compared to strain A7. PBP2x-R61 contains 189 mutations (159 in the soluble region). Positions of all mutations (as compared to the penicillin-sensitive A7) are shown in Figure 1B.

To elucidate this resistance mechanism at an atomic level, we used Modeller software to perform homology modeling of the three-dimensional structure of PBP2x-R61 (see Figure 1A). PBP2x-R61 consists of three domains: an N-terminal domain that folds into an elongated “sugar tong” shape, a central transpeptidase domain (residues 265–619), and a C-terminal domain that is connected via a 26-residue-long flexible loop. The transpeptidase domain, which is homologous to that of other PBPs, contains three conserved amino acid motifs that are in close spatial relationships as part of the active site of the enzyme. These motifs are the S^339^TMK with an active site serine, the S^396^SN triad and the K^548^SG box. In the S^339^TMK motif, the T→A mutation has been extensively verified as a key structural determinant for β-lactam resistance in *S. pneumoniae* [23]. Although this residue is conserved in *S. suis* R61, its neighbor residue has been mutated (M→I). M441 is adjacent to the catalytic S339 and is buried in a small cavity shielded from the active site [24]. We hypothesize that this mutation is somewhat responsible for antibiotic resistance in the R61 isolate.

Although mutations have occurred throughout the entire PBP2x structure, there are two main regions in which the mutations are concentrated: the active site area and the C-terminal domain. The sequence of the C-terminal/transpeptidase domain linker region (residues 619–644) displays 42% variability. We identified PBP2x-R61 residues adjacent to the active site that are potentially less than 5 Å from the cefuroxime molecules (see Figure 1B, marked asterisk below). Generally speaking, mutations of these residues in the binding site could affect antibiotic affinity. In sum, there are 25 potential residues, of which 5 were mutated (E232K, S394A, T489S, T491S and A492V). Interestingly, these mutations are all similar property of side chain.

To further examine drug-resistant mutations, we employed KaKs_Calculator 2.0 [25] to detect regions of positive selection in the PBP2x-R61 sequence. The results show that Ka/Ks (amino acid mutations over synonymous mutations) values in three regions (residues 325–368,526–538 and 684–694) are much higher than 1, meaning that these regions are under strong positive selection pressure imposed by antimicrobials (Figure 1C, 1A). When mapping these three regions onto the 3D structure of PBP2x-R61, we found that they are all in loop regions on the surface of the protein molecule and are adjacent to the drug molecules. Two regions close to the cefuroxime molecule surround the cleft of the catalytic active site. Another region under positive selection is located in the C-terminal domain, which is far away from the active site. However, it is adjacent to the lower cefuroxime molecule. Mutations located in these three regions may affect either enzyme catalysis or dimer stability, or they may reshape the active site through long-range structural perturbations. Thus, these mutations are strong candidates for conferring drug resistance. New experiments will be required to assess whether any of these mutations act as the primary cause of drug resistance or whether they contribute to drug resistance via secondary effects. Based on this analysis, we conclude that the application of drugs is an important force in driving the evolution of PBP2x-R61, which is an essential protein in the acquisition of high-level β-lactam resistance.

In addition to mutations in PBP2x, mutations in PBP2b also lead to high levels of drug resistance in *S. pneumoniae* [26]. We compared PBP2b sequences from R61 and A7 isolates. Mutations occur in a mosaic pattern, just as in PBP2x-R61. Some mutations are homologous to those described in *S. pneumonia*. Full-length PBP2b-R61 carries 30 mutations. A 3D structure of PBP2b-R61 was modeled using Modeller software (see Figure S3A). PBP2b is composed of an N-terminal region and a transpeptidase domain. There are 8 mutations at the N-terminal region of the structure, and all other mutations are within the transpeptidase domain. The central β-sheet of the transpeptidase domain is mostly mutation-free (only one mutation is present). Mutated residues are located mostly on loops or α-helices.

MIC testing revealed that R61 is quinolone-resistant, while strain A7 is quinolone-susceptible. In *Streptococci*, DNA gyrase (composed of 2 subunits, GyrA and GyrB) and topoisomerase IV (made up of ParC and ParE) are the primary targets of quinolone action. A single mutation in the quinolone resistance-determining region (QRDR) of GyrA or ParC can reduce susceptibility to fluoroquinolone [27], [28]. The predicted amino acid sequences of GyrA and ParC in all four quinolone-susceptible isolates (i.e., strains A7, BM407, P1/7 and SC84) revealed no amino acid changes in the QRDRs, implying a high degree of conservation in the two proteins, even among geographically and epidemiologically unrelated strains (Figure S4). However, both GyrA and ParC in the quinolone-resistant strain R61 had mutations. Several critical amino acid positions, which are related to quinolone resistance in other *Streptococci*, also had modifications in the corresponding R61 proteins. Additionally, we found some previously unknown positions that were mutated in the R61 QRDRs, which may provide further clues for understanding the resistance mechanisms in this pathogen.

### Pattern 2: target protection-resistance to tetracycline

Tetracycline resistance is one of the most common bacterial antibiotic resistances. To date more than 40 distinct tetracycline-resistance genes have been described. These genes confer resistance via the following three mechanisms: ribosomal protection, tetracycline efflux and modification of the ribosomal target [29], [30]. We screened for the presence of tetracycline-resistance determinants in R61 and A7 isolates using known *tet* genes. However, because of the low degrees of nucleotide conservation among these known *tet* genes, it is difficult to identify *tet* genes in new strains. Only one CDS (SSUR61_2068) was detected in R61, and no *tet* genes were found in A7, which corresponded with their resistance phenotypes. The best hit for SSUR61_2068 is YP_594556, named *tet*(W), with an E-value of 6×10^−12^ and 34.11% identity. The *tet*(W) gene, which encodes a ribosomal protection protein, has recently been described in a wide range of gram-positive and gram-negative bacteria [31]. However, *tet*(W)-mediated tetracycline resistance has not been previously reported in *S. suis* or in other major streptococcal pathogens, where common determinants are *tet*(M) and *tet*(O) [32]. To further examine the gene responsible for tetracycline resistance, we analyzed the domains in the SSUR61_2068 amino acid sequence. There are three domains in SSUR61_2068: a Tet_M-like domain, an EFTU_α domain and an EFTU_β domain. Tet_M-like domains are found in tetracycline-resistance genes that function through ribosomal protection. Tetracycline binds to ribosomes and changes the ribosomes' conformational states, which disrupts the elongation cycle and stops protein synthesis. Ribosomal protection proteins, which have Tet_M-like domains, are thought to interact with the base of the h34 protein within the ribosome, causing an allosteric disruption of the primary tetracycline binding site(s) and releasing the tetracycline molecules from the ribosome [33], [34].

Holden et al. described the *tet*(M), *tet*(O), and *tet*(L) genes in tetracycline-resistant strain BM407 [16]. *Tet*(M) and *tet*(O) act in ribosomal protection, whereas *tet*(L) works in efflux. Thus, we may conclude that although the BM407 and R61 both have tetracycline resistance, they possess different resistance mechanisms against tetracycline.

### Pattern 3: reduced drug accumulation-resistance to macrolides

We screened for the presence of macrolide-resistance determinants in *S. suis* isolates. Only one CDS (SSUR61_0925) was detected in R61 as a homolog of the resistance gene *mef*(E) (E-value 0.0 and identity 96%). No resistance genes were found in strain A7. Mef(E) is necessary to confer erythromycin and azithromycin resistance [35]. Amelia et al. found that Mef(E) appeared to efflux only 14-membered (erythromycin) and 15-membered (azithromycin) macrolides, and clindamycin was not recognized by the Mef(E) efflux pump [36].

Strain R61 has a clindamycin-resistant phenotype, but the corresponding resistance genes could not be detected. Several studies have reported that the *erm*(B) gene is associated with clindamycin resistance, in addition to macrolide and streptogramin B resistance. The *erm*(B) gene has been frequently discovered as the key clindamycin-resistance gene in a large number of *S. suis* strains, including strain BM407 [16], [37]. To further inspect the absence of *erm* genes in the R61 isolate, we mapped the raw sequencing short reads to all known *erm* gene sequences. No reads clustered, suggesting that there is no *erm* gene in the R61 genome. The current *erm* gene sequences could not help us to detect the R61-resistance gene. This result probably implies that other unknown clindamycin-resistance determinants are present in R61, the confirmation of which requires additional investigations.

### Genome dynamics in *S. suis* strains

**Figure 2:**
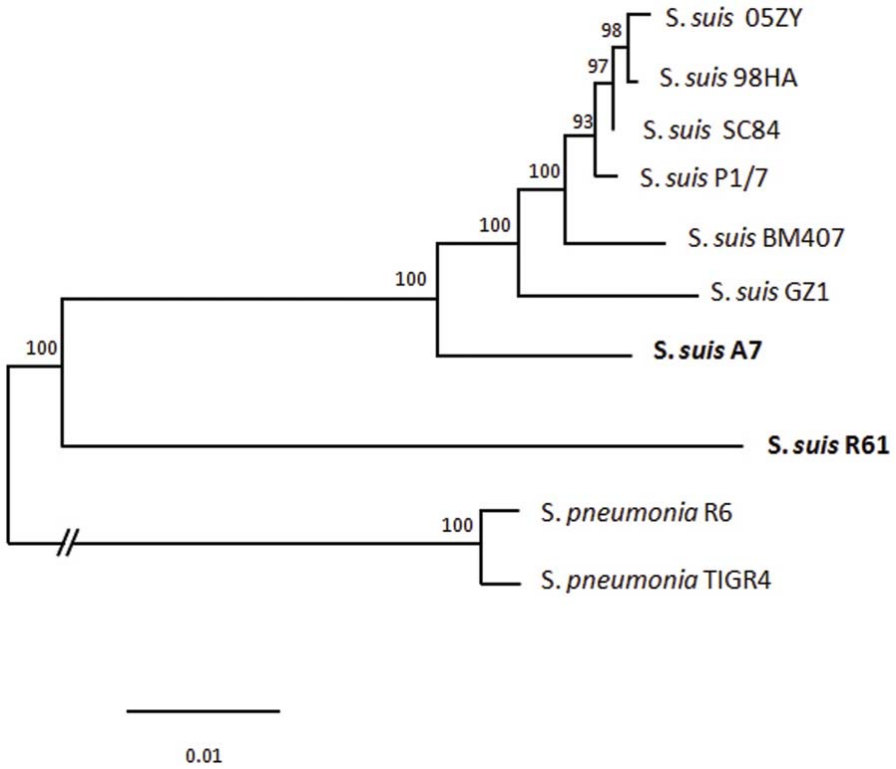
Phylogenetic relationships of S. *suis* strains. A Bayesian phylogenetic tree was obtained for the concatenated sequence alignment of 122 single-copy core genes from each genome of the currently available *S. suis* strains. *S. pneumoniae* TIGR4 and R6 were included as out-groups. The numbers at the branches are posterior probabilities indicating the support for the branch. The bar indicates the number of substitutions per site (1 per 100 sites).

Because of the possibility of horizontal gene transfer, single-gene phylogenies might not reflect the evolutionary history of this species [38], [39]. We used a concatenated DNA sequence obtained by joining 122 single-copy core gene sequences to reconstruct the phylogenetic relationships of *S. suis* strains (see Methods). Bayesian tree revealed that strain R61 is phylogenetically distinct from *S. suis* strains for which genome sequences are currently available (see Figure 2). Moreover, we reconstructed the relationship of all available *S.suis* strains using the MLST typing scheme (http://www.mlst.net/databases/). The results show that the MLST tree has the consistent topological structure with that of the Bayesian tree.

**Figure 3:**
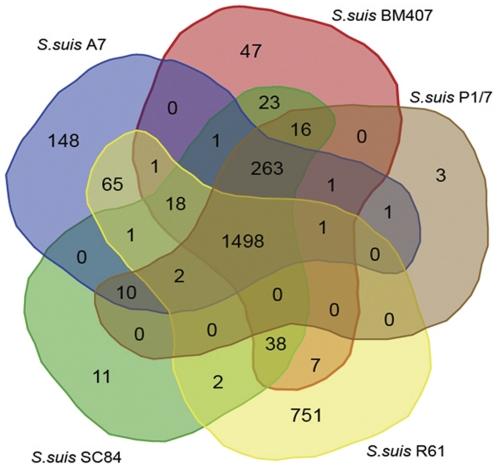
Venn diagram showing the distribution of shared orthologs and strain-special genes between and among *S. suis* strains. Comparative analysis of *S. suis* R61, *S. suis* A7, *S. suis* P/7, *S. suis* BM407 and *S. suis* SC84 strains revealed that there were 1,472 gene clusters that were shared among all five strains. In the case of R61, 1,646 genes of the total 2,346 protein-coding genes aligned in 1,611 clusters. Comparison of the five strains revealed 751 genes that were unique to *S. suis* R61. There were 275 gene clusters that were present in the four other strains but not in R61. Analysis was done using a total of 10,262 genes from the five *S. suis* strains. The Venn diagram was created with web tools provided by the Bioinformatics and Systems Biology Institute of Gent (http://bioinformatics.psb.ugent.be/webtools/Venn/).

Using InParanoid and MultiParanoid software with a cutoff of 50% identity followed by a manual check, CDSs in the five *S. suis* strains were grouped into 1471 clusters (Figure 3). There is a set of 751 predicted CDSs in R61 that were not grouped into ortholog clusters with CDSs from the other four *S. suis* strains (see Figure S5). However, a set of 275 CDSs was shared by all strains but R61. To check for the absence of these CDSs in the R61 genome, we used Maq software [40] to map raw short reads to each sequence of the 275 CDSs respectively. However, this only confirmed that none of these CDSs are present in the R61 genome.

**Figure 4:**
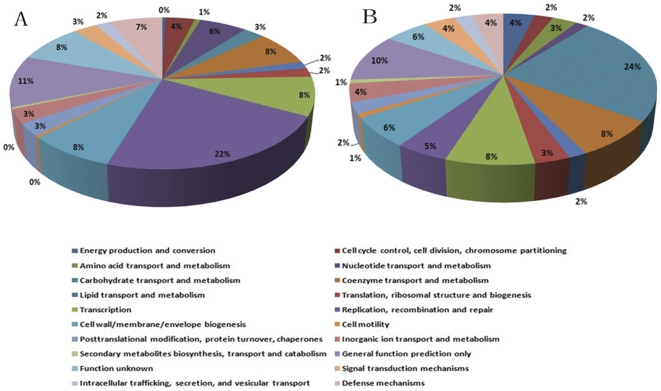
Comparison of COG functional categories between R61 strain-special genes and R61 strain-loss genes. Each colored segment indicates the relative contribution of a functional category as a percentage of total COGs. The color of each COG family is indicated in the figure. (A), COG functional categories of R61 strain-special genes. (B), COG functional categories of R61 strain-loss genes.

The CDSs present in all strains but not in R61 strain are considered as strain-lost genes in the R61 strain. Comparative analysis of five *S. suis* genomes indicates a remarkable degree of gene gain and loss in the genome of strain R61. These strain-specific genes and strain-lost genes in R61 strain both have low percentages of BLAST hits for functional annotation (32.09% in strain-specific genes and 44.87% in strain-lost genes). The functional groups of R61 strain-specific genes are mainly associated with replication, recombination, cell wall/membrane biogenesis and transcription. In contrast, the four highest groups of functional matches for R61 strain-loss genes were identified as carbohydrate transport, replication, coenzyme transport and transcription (Figure 4). We hypothesize that genes related to cell wall/membrane biogenesis and carbohydrate transport might be responsible for drug resistance. It has been clearly demonstrated that bacterial genomes can maintain only a finite number of genes to survive and propagate [41]. Bacterial genomes are prone to eliminate genes that fail to provide a meaningful function to counterbalance gene acquisition. As a result, because of continuous gene gain and loss, bacteria are provided with additional physiological properties that are helpful for exploiting new niches [42]. Comparative analysis also revealed a set of 118 genes that were unique to *S. suis* strain A7, which is apparently higher than the number found in BM407, SC84 and P1/7 strains. This notable diversity might be accounted for by different methods of gene prediction.

To explore the source of these 751 variable genes in the R61 genome, we compared these genes with the NCBI non-redundant database to determine potential horizontal gene transfers from divergent species. BLAST results were filtered to include only different species matches with greater than 30% amino acid identity, E-values less than 1.0×10^−5^ and greater than 70% coverage of the query sequence. Less than half (321/751) of these CDSs had significant BLAST hits (see Table S2). Of the 321 CDSs with hits, a set of 139 CDSs encodes hypothetical proteins, which currently prevents us from a detailed understanding of the significance of these genes. The majority (205/321) of the significant BLAST hits match genomes within *Streptococcus*, suggesting that horizontal gene transfer among closely related species is more frequent than among distant species. Our analysis demonstrates that horizontal gene transfer occurs extensively. More than 20 genera have occurred gene transfers with strain R61. Among the horizontally acquired genes, there are 7 *nus* genes (arranging into one gene cluster), which were identified as coming from *S. uberis* [58]. The *nus* genes cluster was demonstrated to be involved in lantibiotic biosynthesis and regulation in *S. uberis* [43]. Lantibiotics are small antimicrobial peptides that inhibit the growth of a wide range of other gram-positive bacteria. Moreover, lantibiotics are nontoxic to humans and are commonly used as a food preservative [44]. To our knowledge, this is the first report of the existence of the *nus* gene cluster in *S. suis* species.

There are 4 genes identified as drug-resistant genes in the horizontally acquired genes: SSUR61_0925, SSUR61_1068, SSUR61_1983 and SSUR61_2113. SSUR61_0925 was mentioned above as a *mef*(E) gene that confers erythromycin and azithromycin resistance. SSUR61_1068 was a candidate gene responsible for resistance against amikacin, dibekacin, isepamicin, netilmicin, sisomicin and tobramycin. SSUR61_2113 is supposed to be involved in resistance to streptomycin A in strain R61. SSUR61_1983 is a gene encoding β-lactamase_B, which can degrade the β-lactam antibiotic. Acquisition of genes encoding β-lactamase is the most common mechanism of resistance to β-lactam antibiotics in *Enterococcus* species. However, no *Streptococcus* species has yet been described as producing β-lactamase when tested by the chromogenic cephalosporin method [45]. Moreover, genes encoding β-lactamase were also detected in the genome of β-lactam-sensitive strain A7. Clearly, this is not the mechanism of resistance against β-lactam in R61. Thus, our results provide evidence that strain R61 has developed multi-drug-resistance patterns through acquisition of sequences from many divergent lineages.

## Materials and Methods

### Bacterial strains and antimicrobial susceptibility testing

*S. suis* strain R61 was isolated from the lung of a diseased pig in Jiangsu province, China, in 2008, and *S. suis* strain A7 was isolated from the brain of a diseased pig in Hubei province, China, in 2007. Antimicrobial susceptibility testing was performed by assessing the MIC for all isolates using E-test (AB Biodisk, Sweden) according to the manufacturer's recommendations. *Streptococcus pneumoniae* ATCC49619 was used as a control for all antimicrobial susceptibility testing.

### Genome sequencing and assembly

We used a whole-genome shotgun sequencing strategy with Illumina Genome Analyzer sequencing technology. A paired-end sequencing library was constructed with insert sizes of approximately 500 base pairs (bp). We assembled the short reads using SOAPdenovo (http://soap.genomics.org.cn/soapdenovo.html). The de Bruijn graph algorithm used in the software is sensitive to sequencing errors, so we filtered low-quality reads and used only high-quality reads for de novo assembly. To fill the intra-scaffold gaps, we used paired-end information to retrieve read pairs that had one read that was aligned to the contigs and another read that was located in the gap region. With this information, we did a local assembly for the collected reads. Then, these scaffolds were ordered relative to the genome of *S. suis* strain 05ZYH33 (deposited in the NCBI database; GenBank accession number CP000407) using a combination of the MUMmer 3 [46] and BLAST [47] programs, and an independent assembly was constructed with Phred/Phrap/Consed software [48]–[50]. Gaps between any remaining internal scaffolds were closed by PCR and edited with Consed. Potential misassemblies were corrected with PCR amplification and long-read sequencing.

Sequences and annotations of the *Streptococcus suis* strains R61 and A7 genomes were deposited in the NCBI database under accession numbers AEYY01000000 and CP002570, respectively.

### Genome annotation and ortholog analysis

Initial open reading frames (ORFs) predictions were performed using Glimmer3 [51] and Genemarks.hmm [52]. The two predictions were amalgamated. All known protein sequences from other *S. suis* strains (determined from NCBI databases) were compared to our entire DNA sequences using TBLASTN to identify any previously missed coding sequences. All putative ORFs were translated into their respective amino acid sequences and subjected to BLASTP with a set expected score of 1×10^−6^, in the non-redundant protein database (nr, downloaded from NCBI on October 5, 2010). Those ORFs without BLAST hits against any other protein were automatically annotated as “hypothetical proteins”. ORFs were grouped into homologous clusters using InParanoid4 [53], [54] and MultiParanoid [55] with the default parameters. InParanoid4 utilizes pairwise similarity scores, which are calculated using BLAST, to construct orthology groups between two species. MultiParanoid applies a clustering algorithm to merge multiple pairwise ortholog groups from InParanoid into multi-species ortholog clusters. Each cluster was manually inspected, and paralogs were kept out. tRNAs and rRNAs were identified using tRNAscan-SE [56] and RNAmmer1.2 [57], respectively. IS elements were identified using ISFinder [58].

### Phylogenetic analysis

To gain a better understanding of genome evolution in *S. suis* strains, six additional completely sequenced *S. suis* genome sequences were obtained from NCBI (http://www.ncbi.nlm.nih.gov). Additionally, we selected two closely related *S. pneumoniae* genomes to serve as outgroups. We used 122 single-copy core genes with nearly identical length and exactly one member in each of the compared strains for phylogenetic analysis. Sequence alignments of these genes were concatenated into a large alignment of 102,365 nucleotides. A phylogenetic tree was reconstructed using MrBayes 3 [59], [60] (200,000 generations sampled every 100 generations with a gamma distribution model and invariant class).

### Identification of antibiotic-resistance determinants

Antibiotic-resistance genes were identified and characterized mainly by the following strategies: the amino acid sequence of each gene was compared with the ARDB with an expected score cutoff of 1×10^−5^. Matches were screened such that only hits with 35% or greater identity were accepted as candidate resistance genes. To further explore resistance mechanisms from a structural viewpoint, we used Modeller [61] to examine the homology of three-dimensional protein structures with the structures of antibiotic-resistance proteins. This software can implement comparative protein structure modeling with known related structures by satisfying spatial restraints. The unstable conformation of loop regions was further optimized by the loop model class in Modeller.

We employed the software KaKs_Calculator 2.0 to detect the positive selection regions, which is a toolbox that calculates nonsynonymous (Ka) and synonymous (Ks) substitution rates of two compared DNA sequences by means of various models. In our study, the input file is aligned DNA sequences of PBP2x-R61 and PBP2x-A7. We used a sliding window of 30 bp (step size 6 bp) along the PBP2x-R61 DNA sequence to calculate the Ka/Ks value of each 30 bp region by YN model. The value indicates selective pressures on different regions.

### Horizontal gene transfer

Genes present in only one strain were considered strain-specific genes. In our study, if InParanoid4 did not group the predicted amino acid sequence of a gene into a homologous cluster with protein sequences from any other studied strains, the gene was considered a strain-specific gene. Strain-specific genes from each species were compared with the NCBI non-redundant database using BLASTP with the expected value cutoff set at 1.0×10^−5^ to identify homologs in other species. Matches were screened such that only hits with 30% or greater identity and 70% or greater coverage were accepted as potential sources of horizontal gene transfer.

## Supporting Information

Figure S1 **Schematic circular diagram of the** ***S. suis*** **A7 genome.** The circles represent from the outside: circle 1, DNA base position (kb); circle 2, protein-coding regions transcribed clockwise; circle 3, protein-coding regions transcribed anticlockwise; circle 4, protein-coding regions coloured according to their functional classification into the Clusters of Orthologous Groups of proteins; circle 5, tRNA, rRNA and miscellaneous RNA; circle 6, G/C skew plotted using a 2-kb window and a 0.2-kb sliding step; circle 7, G+C content plotted using a 2-kb window and a 0.2-kb sliding step.
(TIF)

Figure S2 **Synteny between the chromosome of** ***S. suis*** **A7 and the assembled contigs of** ***S. suis*** **R61.** The X-Y plot is composed of dots forming syntenic regions between both genomes. The dots represent predicted *S. suis* R61 proteins having an orthologue in the genome of *S. suis* A7 with co-ordinates corresponding to the position of the respective coding region in *S. suis* A7 genome sequence and indicated in MB. Red dots mean positive corresponding, whereas blue dots indicate opposite corresponding.
(TIF)

Figure S3 **Comparison of PBP2b-R61 and PBP2b-A7; structure of PBP2b from drug-resistant strain R61.** The enzyme is composed of two parts: N-terminal region and the transpeptidase domain. Yellow spheres represent locations of mutations. It is of note that the central β-sheet of transpeptidase domain is mostly mutation-free (only one mutation happened). Mutated residues are located mostly on loops or α-helices.
(TIF)

Figure S4 **Comparison of PBP2b-R61 and PBP2b-A7; sequence alignment and secondary structure assignment.** Sequence alignment of PPB2b from strain A7 and strain R61. Conserved mutations are represented in blue, while non-conserved changes are shown in red. Secondary structural elements referring to the R61 PBP2b structure are shown as red cylinders (α-helices), blue rectangles(β-sheets).
(TIF)

Figure S5 **Amino acid sequence alignment of the quinolone resistance-determining regions (QRDRs) of GyrA and ParC in** ***S. suis*** **strains.** Except R61, all strains are drug-susceptible. Amino acids critical for quinolone resistance are marked with an arrowhead. Three mutations exist in QRDR of GyrA-R61, with two critical. Only one of the two critical amino acids mutates in QRDR of ParC-R61.
(TIF)

Figure S6 **Strain-special genes and horizontally acquired genes in the** ***S. suis*** **R61 genome.** We concatenated 53 assembled contigs into large nucleotides to draw the circular diagram of R61 genome. The whole length of the concatenated nucleotides is 2,390,900 bp. The total length of strain-special genes is 486,281 bp (accounting for ∼20% of the assembled R61 genome). The circles represent from the outside: circle 1, DNA base position (kb); circle 2, strain-special genes transcribed clockwise; circle 3, strain-special genes transcribed anticlockwise; circle 4, horizontally acquired genes.
(TIF)

Table S1 **Antibiotic resistance determinants detected in S.** ***suis*** **strains by searching ARDB.** (http://ardb.cbcb.umd.edu/index.html).
(DOC)

Table S2 **Complete list horizontally acquired genes in R61.**
(XLSX)
