# Supplementary figures and images for: Comparative Genomics Study of Multi-Drug-Resistance Mechanisms in the Antibiotic-Resistant Streptococcus suis R61 Strain
Source: PLoS One. 2011 Sep 26;6(9):e24988. doi: 10.1371/journal.pone.0024988 (PMC3180280; doi:10.1371/journal.pone.0024988)

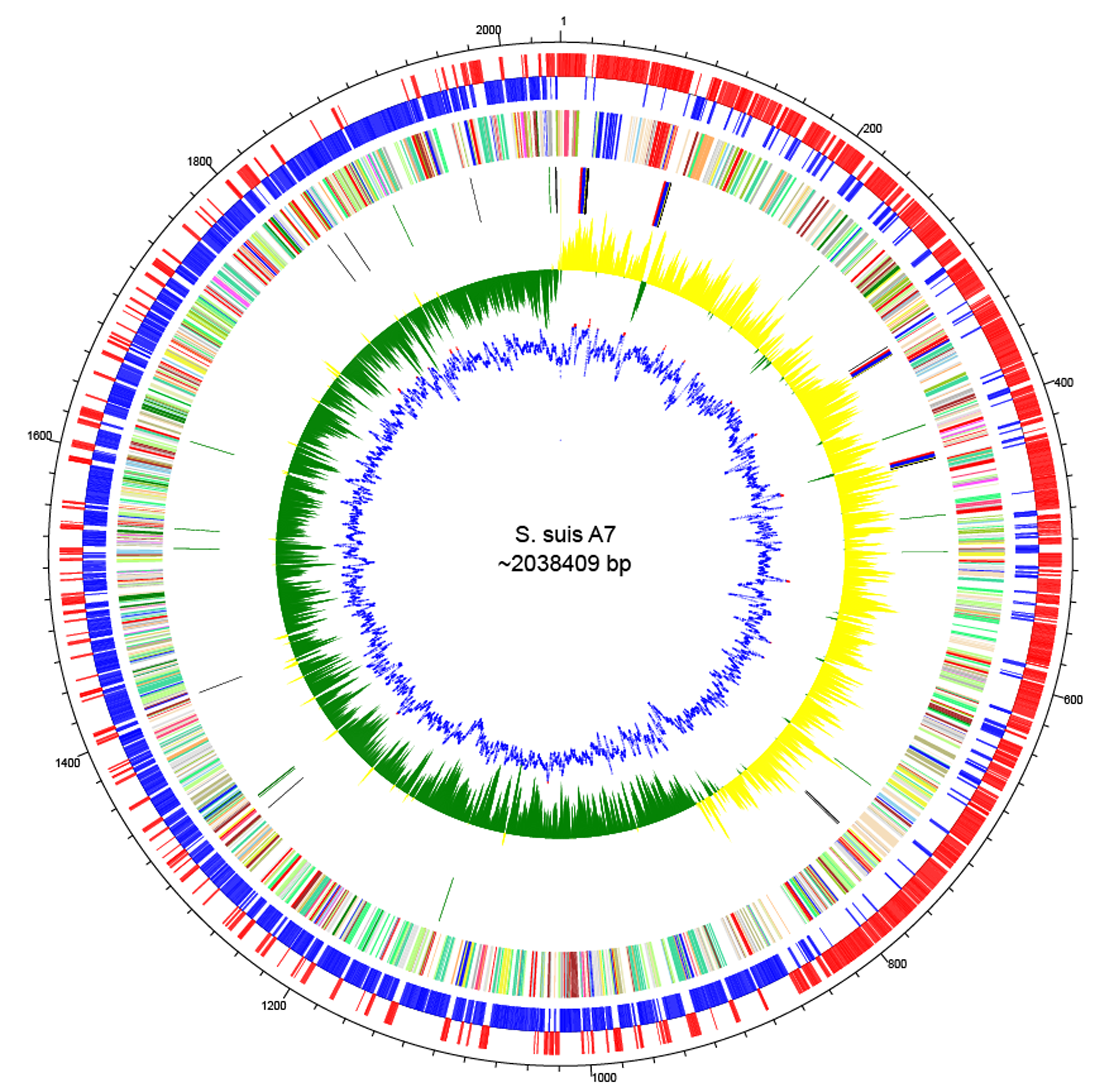

Supplement: Figure S1 — Schematic circular diagram of the S. suis A7 genome. The circles represent from the outside: circle 1, DNA base position (kb); circle 2, protein-coding regions transcribed clockwise; circle 3, protein-coding regions transcribed anticlockwise; circle 4, protein-coding regions coloured according to their functional classification into the Clusters of Orthologous Groups of proteins; circle 5, tRNA, rRNA and miscellaneous RNA; circle 6, G/C skew plotted using a 2-kb window and a 0.2-kb sliding step; circle 7, G+C content plotted using a 2-kb window and a 0.2-kb sliding step. (TIF) [file pone.0024988.s001.tif]

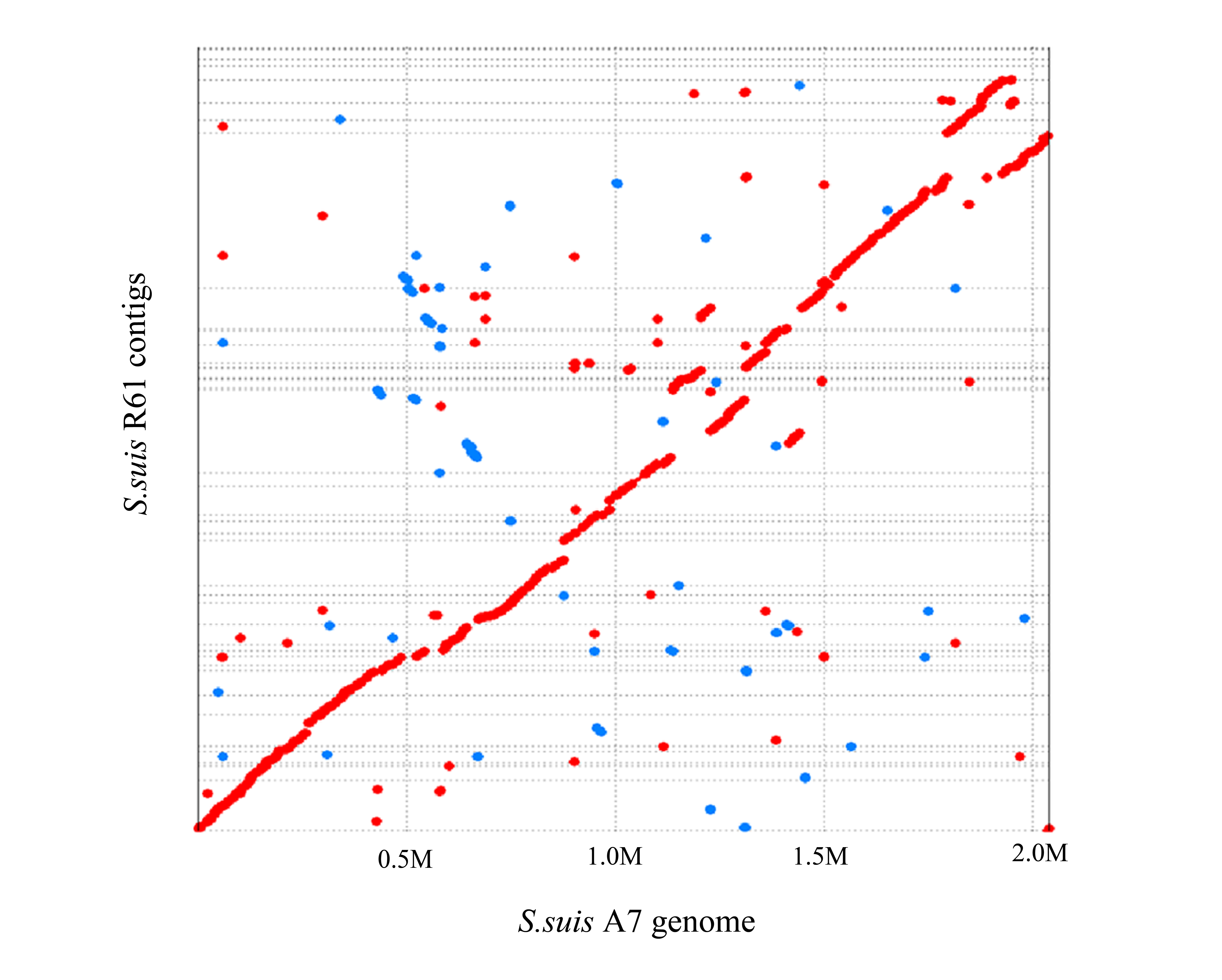

Supplement: Figure S2 — Synteny between the chromosome of S. suis A7 and the assembled contigs of S. suis R61. The X-Y plot is composed of dots forming syntenic regions between both genomes. The dots represent predicted S. suis R61 proteins having an orthologue in the genome of S. suis A7 with co-ordinates corresponding to the position of the respective coding region in S. suis A7 genome sequence and indicated in MB. Red dots mean positive corresponding, whereas blue dots indicate opposite corresponding. (TIF) [file pone.0024988.s002.tif]

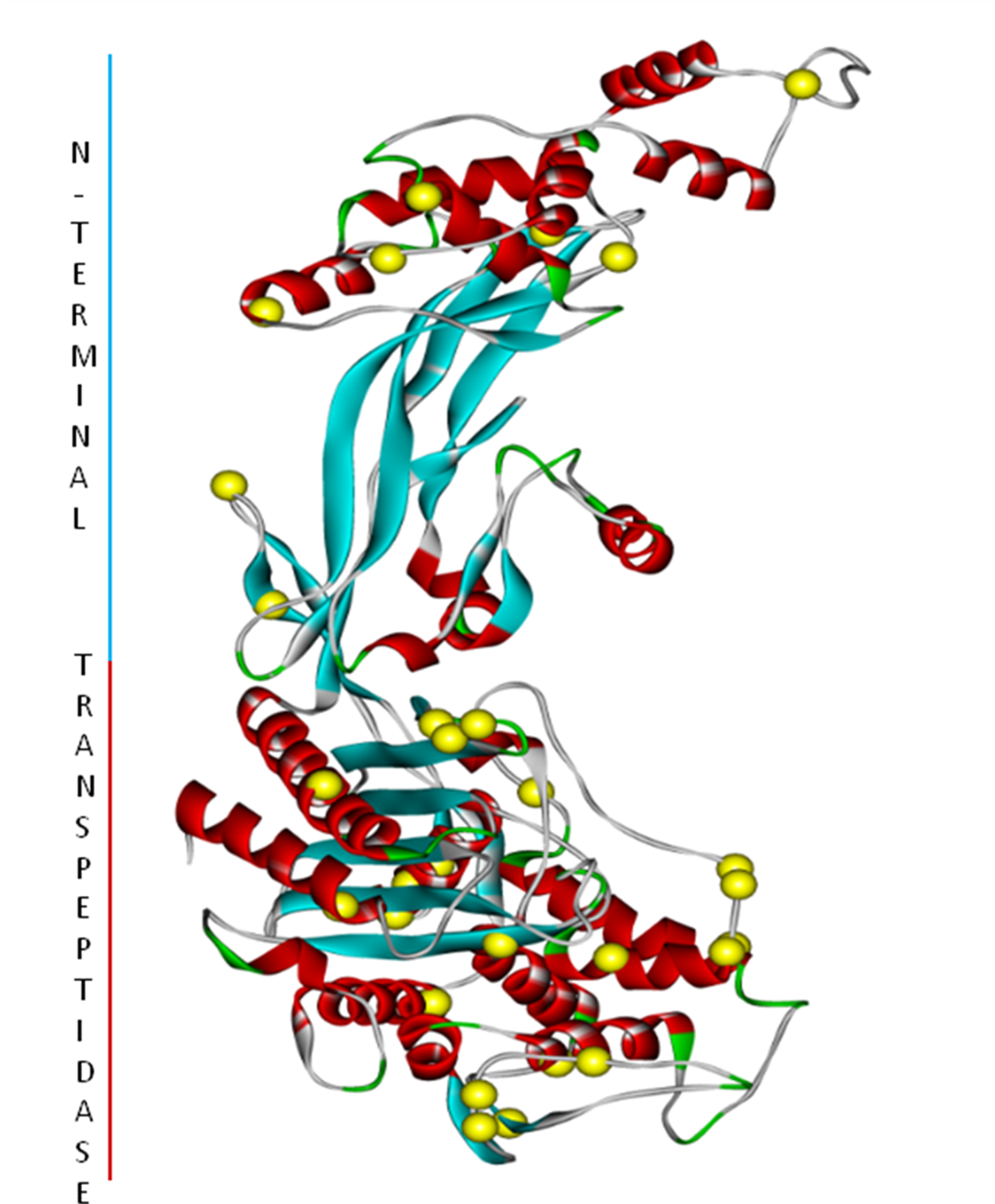

Supplement: Figure S3 — Comparison of PBP2b-R61 and PBP2b-A7; structure of PBP2b from drug-resistant strain R61. The enzyme is composed of two parts: N-terminal region and the transpeptidase domain. Yellow spheres represent locations of mutations. It is of note that the central β-sheet of transpeptidase domain is mostly mutation-free (only one mutation happened). Mutated residues are located mostly on loops or α-helices. (TIF) [file pone.0024988.s003.tif]

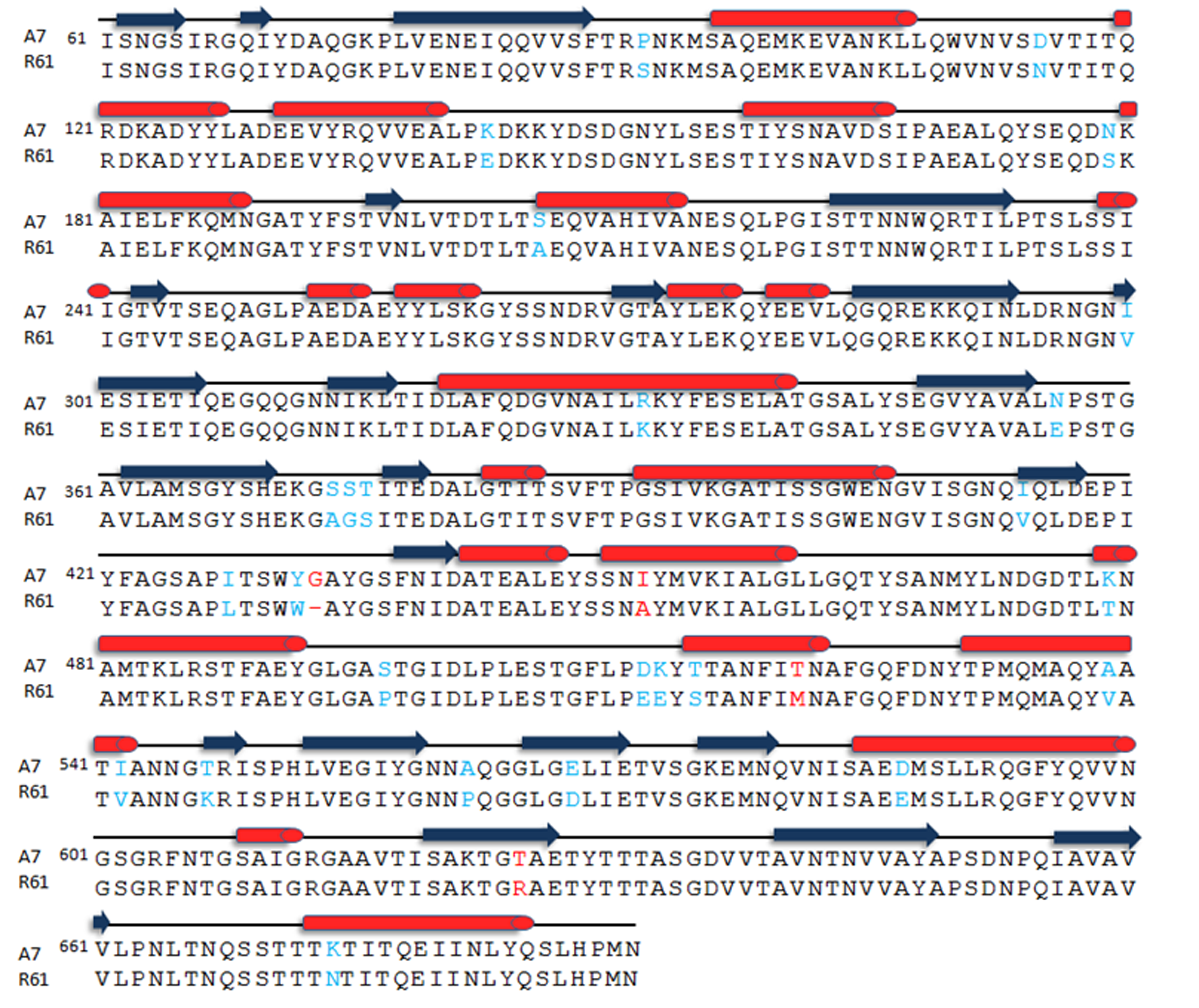

Supplement: Figure S4 — Comparison of PBP2b-R61 and PBP2b-A7; sequence alignment and secondary structure assignment. Sequence alignment of PPB2b from strain A7 and strain R61. Conserved mutations are represented in blue, while non-conserved changes are shown in red. Secondary structural elements referring to the R61 PBP2b structure are shown as red cylinders (α-helices), blue rectangles(β-sheets). (TIF) [file pone.0024988.s004.tif]

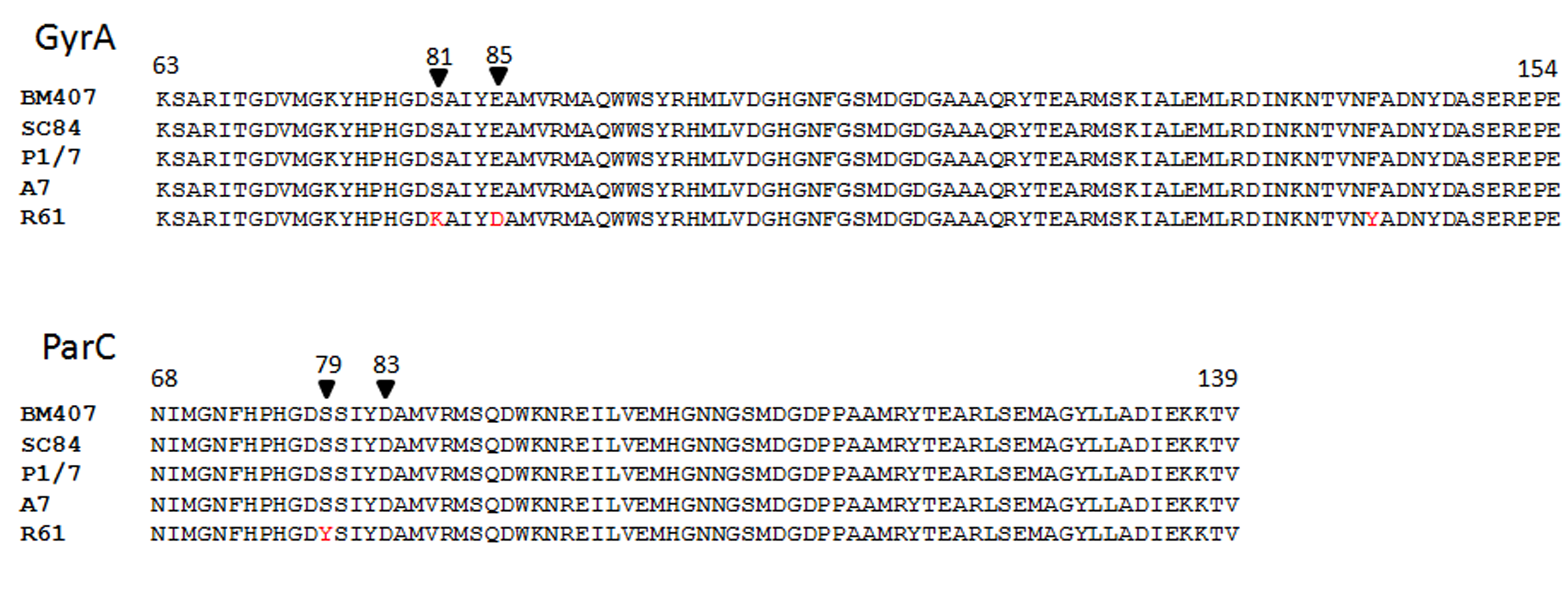

Supplement: Figure S5 — Amino acid sequence alignment of the quinolone resistance-determining regions (QRDRs) of GyrA and ParC in S. suis strains. Except R61, all strains are drug-susceptible. Amino acids critical for quinolone resistance are marked with an arrowhead. Three mutations exist in QRDR of GyrA-R61, with two critical. Only one of the two critical amino acids mutates in QRDR of ParC-R61. (TIF) [file pone.0024988.s005.tif]

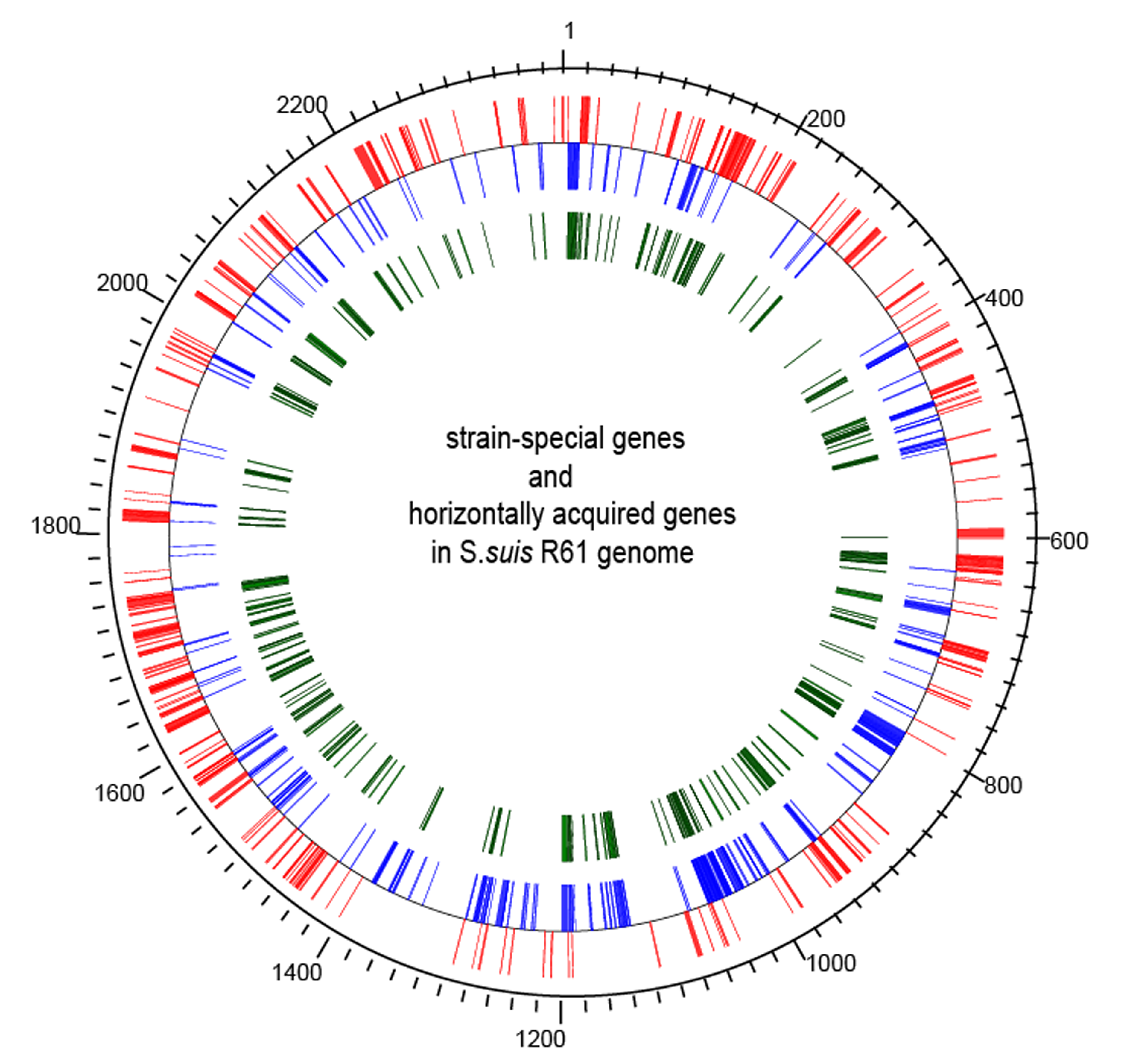

Supplement: Figure S6 — Strain-special genes and horizontally acquired genes in the S. suis R61 genome. We concatenated 53 assembled contigs into large nucleotides to draw the circular diagram of R61 genome. The whole length of the concatenated nucleotides is 2,390,900 bp. The total length of strain-special genes is 486,281 bp (accounting for ∼20% of the assembled R61 genome). The circles represent from the outside: circle 1, DNA base position (kb); circle 2, strain-special genes transcribed clockwise; circle 3, strain-special genes transcribed anticlockwise; circle 4, horizontally acquired genes. (TIF) [file pone.0024988.s006.tif]
