# Supplementary material for: Comparative Genomics Study of Multi-Drug-Resistance Mechanisms in the Antibiotic-Resistant Streptococcus suis R61 Strain
Source: PLoS One. 2011 Sep 26;6(9):e24988. doi: 10.1371/journal.pone.0024988 (PMC3180280; doi:10.1371/journal.pone.0024988)
Supplement: Table S1 — Antibiotic resistance determinants detected in S. suis strains by searching ARDB. (http://ardb.cbcb.umd.edu/index.html). (DOC) [file pone.0024988.s007.doc]

Table S1. Antibiotic resistance determinants detected in S. *suis* strains by searching ARDB (http://ardb.cbcb.umd.edu/index.html)

| **Strains** | **Resistance** | **Candidate gene** | **Best hit** | **E-value** | **Identity**  **%** | **Hit**  **Length** | **HSP Length** |
| --- | --- | --- | --- | --- | --- | --- | --- |
| **R61** | Bacitracin | orf1796 | YP_001455939 | 6e-40 | 36.20 | 272 | 279 |
|  | Macrolide | orf0948 | YP_001396002 | 0.0 | 89.63 | 408 | 405 |
|  | Amikacin  dibekacin isepamicin netilmicin sisomicin tobramycin | orf1194 | CAL22895 | 0.0 | 97.49 | 479 | 479 |
|  | Ciprofloxacin  Norfloxacin | orf2145 | EDK62636 | 1e-123 | 53.55 | 399 | 394 |
|  | Streptomycin | orf0951 | ABP57330 | 1e-104 | 61.89 | 288 | 286 |
|  | Streptomycin_a | orf0545  orf2158 | YP_001038094  AAA86871 | 5e-06 1e-09 | 51.22  46.00 | 211 212 | 41  50 |
| **A7** | Bacitracin | orf1728 | YP_001455939 | 1e-24 | 37.84 | 272 | 185 |
| **P1/7** | Bacitracin | YP_003027507 | YP_001455939 | 8e-40 | 36.23 | 272 | 276 |
| **BM407** | Bacitracin | YP_003028699 | YP_001455939 | 8e-40 | 36.23 | 272 | 276 |
|  | Lincosamide  Macrolide  Streptogramin_b | YP_003028697 | CAJ21502 | 1e-140 | 99.59 | 245 | 245 |
|  | Tetracycline | YP_003028728  YP_003028727  YP_003028699 | AAL92527  ABB70050  EDP26737 | 0.0  0.0  0.0 | 97.82  99.53  97.81 | 458  639  639 | 458  639  639 |
| **SC84** | Bacitracin | YP_003024841 | YP_001455939 | 8e-40 | 36.23 | 272 | 276 |
|  | Tetracycline | YP_003024841 | ABB70050 | 0.0 | 100.00 | 639 | 639 |
